# Molecular analyses indicate profuse bacterial diversity in primary and post- treatment endodontic infections within a cohort from the United Arab Emirates-A preliminary study

**DOI:** 10.1371/journal.pone.0305537

**Published:** 2024-07-15

**Authors:** Sheela B. Abraham, Farah Al-Marzooq, Lakshman Samaranayake, Rifat Akram Hamoudi, Wan Harun Himratul-Aznita, Hany Mohamed Aly Ahmed

**Affiliations:** 1 Department of Restorative Dentistry, College of Dental Medicine, University of Sharjah, Sharjah, United Arab Emirates; 2 Department of Microbiology and Immunology, College of Medicine and Health Sciences, UAE University, Al Ain, UAE; 3 Faculty of Dentistry, University of Hong Kong, Hong Kong; 4 Faculty of Dentistry, Chulalongkorn University, Bangkok, Thailand; 5 Research Institute for Medical and Health Sciences, College of Medicine, University of Sharjah, Sharjah, United Arab Emirates; 6 Division of Surgery and Interventional Sciences, University College London, London, United Kingdom; 7 ASPIRE Precision Medicine Research Institute Abu Dhabi, University of Sharjah, Sharjah, United Arab Emirates; 8 Department of Oral & Craniofacial Sciences, Faculty of Dentistry, University of Malaya, Kuala Lumpur, Malaya; 9 Department of Restorative Dentistry, Faculty of Dentistry, University of Malaya, Kuala Lumpur, Malaya; University of Puthisastra, CAMBODIA

## Abstract

**Objective:**

Endodontic microbiota appears to undergo evolutionary changes during disease progression from inflammation to necrosis and post-treatment. The aim of this study was to compare microbiome composition and diversity in primary and post-treatment endodontic infections from a cohort of patients from the UAE.

**Design:**

Intracanal samples were collected from primarily infected (n = 10) and post-treatment infected (n = 10) root canals of human teeth using sterile paper points. Bacterial DNA was amplified from seven hypervariable regions (V2–V4 and V6–V9) of the 16S rRNA gene, then sequenced using next-generation sequencing technology. The data was analyzed using appropriate bioinformatic tools.

**Results:**

Analyses of all the samples revealed eight major bacterial phyla, 112 genera and 260 species. *Firmicutes* was the most representative phylum in both groups and was significantly more abundant in the post-treatment (54.4%) than in primary (32.2%) infections (p>0.05). A total of 260 operational taxonomic units (OTUs) were identified, of which 126 (48.5%) were shared between the groups, while 83 (31.9%) and 51 (19.6%) disparate species were isolated from primary and post-treatment infections, respectively. A significant difference in beta, but not alpha diversity was noted using several different indices (p< 0.05). Differential abundance analysis indicated that, *Prevotella maculosa*, *Streptococcus constellatus*, *Novosphigobium sediminicola* and *Anaerococcus octavius* were more abundant in primary infections while *Enterrococcus faecalis*, *Bifidobacterium dentium*, *Olsenella profusa* and *Actinomyces dentalis* were more abundant in post-treatment infections (p <0.05).

**Conclusion:**

Significant differences in the microbiome composition and diversity in primary and post-treatment endodontic infections were noted in our UAE cohort. Such compositional differences of microbiota at various stages of infection could be due to both intrinsic and extrinsic factors impacting the root canal ecosystem during disease progression, as well as during their therapeutic management. Identification of the key microbiota in primarily and secondarily infected root canals can guide in the management of these infections.

## Introduction

Dysbiotic oral microbiota are the prime trigger of oral diseases such as dental caries and periodontal disease that may culminate in pulpal and endodontic infections [1]. Indeed, the composition of the bacterial communities that initially colonize the pulpal space is largely dependent on the specific ecological conditions that precipitate the infection [2]. For instance, microbes that initially invade and colonize the pulp space can cause primary endodontic infections characterized by a predominantly polymicrobial, anaerobic flora [3]. On the other hand, secondary (persistent) infection ensues when microbes of a post-primary or secondary infection which resisted intracanal antimicrobial interventions survive under adverse conditions with nutrient deprivation in `treated`canals [4].

It is known that over 460 bacterial taxa are associated with infected root canals [5]. Thus, the composition of microbiota in endodontic infections is highly complex with many yet to be deciphered [6]. Indeed, novel molecular identification techniques such as next generation sequencing (NGS), and pyrosequencing have helped the speciation of most, hitherto unknown microbiota in such infections, particularly, those in apical peri-radicular diseases [7]. This technique enables a large number of reads in a single run providing increased sampling depth when compared with other traditional techniques [8, 9]. In particular, NGS technology permits the hitherto unknown detection of low abundant genera [10].

Earlier molecular studies comparing the endodontic microbiota of patients from varying geographic locales revealed significant differences in the prevalence of candidate pathogens [11, 12]. This is also evident from a review by Shin et al., (2018) who profiled the endodontic microbiota of populations from Brazil, United States, Netherlands, Sudan, South Korea, Estonia, Greece, and Turkey [7]. Presently, there is no equivalent data of the endodontic microbiota of United Arab Emirates (UAE) and Gulf populations.

Some of these studies have revealed the identity of root canal microbiota in extracted teeth with primary and secondary apical periodontitis and noted that the most abundant species in dentine and root canal samples was the anaerobe *Fusobacterium nucleatum* [13]. Additionally, they also provided evidence of microbial interactions that were specific to the type of infection. Recently, Buonavoglia et al., (2021) assessed the microbiota in endo-perio lesions and suggested that there may be disparate eco-niches even within the same endodontic canal system displaying different microbes [14].

Due to the fact that endodontic flora from disparate locales differ and as there is sparsity of information from the Gulf regions, the main objective of the present study, therefore, was to compare and contrast the composition of the endodontic microbiota of primary and post-treatment infected root canal systems and examine their diversity, using NGS and bacterial 16S rRNA typing methodology.

## Materials and methods

### Ethical approval

Patients who attended the endodontic clinic at the University of Sharjah Dental Hospital were invited to participate in the study. The study was approved by the Research Ethics Committee of the University of Sharjah (No. REC-18-05-30-01). An informed written consent was obtained from each patient prior to enrolment in the study.

### Sample size calculation and patients’ selection

The sample size was calculated based on previous NGS literature. In a systematic review of 18 microbiome studies by Manoil et al., (2020), seven studies cited, compared primary and post-treatment infections and the mean sample size was 28 [15]. Based on the time available for patient recruitment and resources for sample processing and analysis, it was proposed to recruit 20 patients for the study:10 samples each for primary and post-treatment infection group.

### Inclusion and exclusion criteria

Patients with irreversible pulpitis (n = 10) who experienced spontaneous pain or long- lasting moderate to severe pain during a cold test with Endo-Ice (1,1,1,2 tetrafluoroethane; Hygienic Corp, Akron, OH) and a bleeding coronal pulp on access opening and apical periodontitis on radiographic assessment were included in the study. For post-treatment cases, adult patients with a root canal treated tooth, done at least 12 months ago showing radiographic evidence of apical periodontitis were included. Patients’ gender, age, tooth type, clinical symptoms, clinical findings of dental examination such as tenderness to percussion, presence/absence of periapical radiolucency and the radiographic quality of the root-canal filling were recorded. Coronal restorations were categorized as defective when open margins, fractured restorations, or recurrent carious lesion were detected.

Patients in whom rubber dam could not be applied, patients with generalized periodontal disease, with edematous or fibrous gingiva, periodontal pockets more than 3 mm, horizontal or vertical bone loss, advanced mobility, presence of intra canal posts and radicular fractures were excluded from the study. Patients younger than 18 years of age, those with systemic diseases, cancer, immunodeficiency disorder and patients who received antibiotics three months prior to sampling were also excluded. All samples were taken by a single operator using an identical technique over a period of six months from January to June 2019.

### Sample collection and DNA extraction

A total of 20 infected intracanal samples were collected from 10 primary (F) and 10 post-treatment (S) cases by a single trained dental specialist over a period of six months. The protocol for sample collection was similar to that described by Pinheiro et al., [16]. Sampling was performed following thorough disinfection of the area around the tooth to ensure sterility and avoid contamination. Briefly, the tooth surface was cleaned with pumice and isolated with a rubber dam. The tooth and the operating field were disinfected with a 30% hydrogen peroxide solution (H2O2), followed by 2.5% sodium hypochlorite solution (NaOCl). The surface was inactivated with 10% sodium thiosulfate for 1 min. To ensure decontamination, random samples of the specimen surface were taken and processed using qPCR analysis using universal primers.

The pulp was accessed with sterile high-speed diamond burs (Brasseler) and was extirpated using sterile saline for intracanal irrigation. Sterile #25 paper points (Dentsply Sirona, Endodontics, USA) were used to soak up the fluid in the canal, after being kept in place for a minute. A single root canal was always sampled to confine the microbial evaluation to a single ecological environment. In multi-rooted teeth, the root with the periapical lesion was selected. If all roots had periapical lesions, the widest canal was sampled. In post-treatment cases, no solvent was used to remove gutta percha and the gutta percha was removed by sterile Gates Glidden burs #3 and #4 (Dentsply Sirona Endodontics, USA) or the ProTaper retreatment files, sizes D1, D2 and D3 (Dentsply Sirona Endodontics, USA) on an X smart Endo Motor (Dentsply Sirona Endodontics, USA). The apical material was retrieved using stainless steel K- and/or Hedström files sizes 25 (Dentsply Sirona Endodontics, USA). After irrigating with saline, samples were collected with paper points as mentioned above. Negative control using sterile paper points, not applied to the root canal was used in parallel to test for contaminants in the paper points. The collected samples were placed in an Eppendorf centrifuge tube (1.5 ml) containing 300 μl of phosphate buffered saline (PBS) and immediately frozen at -20°C until DNA extraction.

DNA extraction was performed using MasterPure^™^ Complete DNA and RNA Purification (Epicenter, USA), following the manufacturer’s instructions. The quality and the quantity of the DNA was evaluated by the absorbance measurements at A260/280 using Colibri Microvolume Spectrometer (Titertek-Berthold Detection Systems GmbH, Germany). Extracted DNA samples were considered pure if the A260/280 ratio was higher than 1.8.

### Amplification of 16S rRNA hypervariable regions and library preparation

Ion 16S metagenomics kit (Thermo Fisher Scientific, USA) was used for selectively amplifying the corresponding hypervariable regions of the bacterial 16S rRNA gene. 16S rRNA amplicons were prepared using two multiplex PCR assays. Pool 1 contained primers targeting the V2-4-8 hypervariable regions whereas pool 2 contained those targeting the V3–6, 7–9 hypervariable regions [17]. For each sample, two reactions were prepared (one with each set of primers), using ’water’ as negative control and ’diluted *E*. *coli* DNA’ as positive control. After PCR amplification, equal volumes (20 μl) of PCR products from pools 1 and 2 of each sample were combined and purified using the AmpureXP beads (Beckman Coulter, Brea, CA, USA) according to the manufacturer’s instructions followed by DNA quantification using the Qubit Fluorometer with the Qubit dsDNA HS Assay Kit (Thermo Fisher Scientific, Warrington, England). DNA library was prepared using Ion Plus Fragment Library Kit (Thermo Fisher Scientific, Austin, TX, USA) and Ion Xpress Barcodes Adapters 1–16 KitTM (Life Technologies, Carlsbad, CA, USA) according to the manufacturer’s protocol. Thus, the prepared DNA library was purified with AMPure XP beads (Beckman Coulter, Brea, CA, USA). After a five-cycle PCR reaction and purification with AMPure XP beads (Beckman Coulter, Brea, CA, USA), library concentrations were determined using the Qubit Fluorometer (Thermo Fisher Scientific, Warrington, England).

### Next-generation sequencing

Ion 520 kit (Thermo Fisher Scientific, Austin, TX, USA) for the Ion S5 workflow were used in this study. The prepared libraries were diluted to 10 pM and pooled equally with twenty individual samples per pool and were clonally amplified using emulsion PCR on Ion One Touch-2 instruments (OT2) followed by enrichment on Ion One Touch ES. Then, prepared libraries were sequenced on Ion S5 XL Semiconductor sequencer using Ion 520 chip.

### Bioinformatic analyses

After sequencing, individual sequence-reads were filtered to remove low-quality sequences. Quality control of sequencing reads retained sequences with a length between 140 and 400 bp. All quality-approved, trimmed, and filtered data were exported as bam files. Chimeric sequences were automatically identified and removed. Operational taxonomic unit (OTU) clustering was performed using the Microbiome Analyst program [18, 19], which runs Quantitative Insights into Microbial Ecology (QIIME).

Further sequence analysis was performed by Uparse software [20], using all the effective tags. Sequences with >97% similarity were assigned to the same OTU. Representative sequence for each OTU was screened for further annotation. Microbiome Analyst was used to visualize the taxonomic composition of community at various taxonomic levels namely phylum, class, order, family, genus, and species level. OTUs abundance information was normalized using a standard of sequence number corresponding to the sample with least sequences. All further analyses of alpha and beta diversity were performed based on this normalized data.

### Diversity analysis

Alpha and beta diversity graphics created by QIIME software were exported from the Microbiome Analyst software to calculate downstream diversity measures using alpha and beta diversity metrics. Shannon and Simpson diversity indices, Chao1-type estimator for diversity from abundance data, ACE (Abundance-based Coverage Estimator) were used to estimate microbiota α-diversity, richness, and evenness. For β-diversity (between groups comparison), Bray–Curtis and Jaccard distance matrices were used to assess the dissimilarity of samples and visualized through principal coordinate analysis (PCoA) and dendrograms. To test whether primary (F) and post-treatment (S) groups samples significantly differed in their microbiota, the statistical significance of the clustering pattern in ordination plots of beta diversity was evaluated using Permutational ANOVA (PERMANOVA). Dendrograms with hierarchical clustering were also used to visualize the relation among samples from various groups based on beta diversity.

### Statistical analysis

SPSS Statistics 28 software (IBM SPSS^®^ Statistics, Armonk, NY, USA) was used for statistical analysis. To evaluate the significant differences between primary and post-treatment samples, continuous variables were compared using the t-test. A p-value of ≤0.05 was considered significant. The Microbiome Analyst 2.0 platform (McGill, Canada) was used to determine linear discriminant analysis (LDA) effect size (LEfSe) to perform differential abundance analysis.

Venn diagram was generated to show the shared and unique OTUs between primary and post-treatment samples, based on the occurrence of OTUs in a group regardless of their relative abundance using the Venny bioinformatics tool (version 2.1). Heatmaps were constructed using R version 4.0.1 (package: gplots; function: heatmap.2).

## Results

### Taxonomic identification and relative abundance

A total of 5.3 million filtered paired end reads of 16S rRNA sequence, clustered into 260 OTUs were obtained by analyzing the 20 samples. There were 209 OTUs and 176 OTUs detected from the samples of primary infection and the post-treatment groups, respectively.

**Fig 1:**
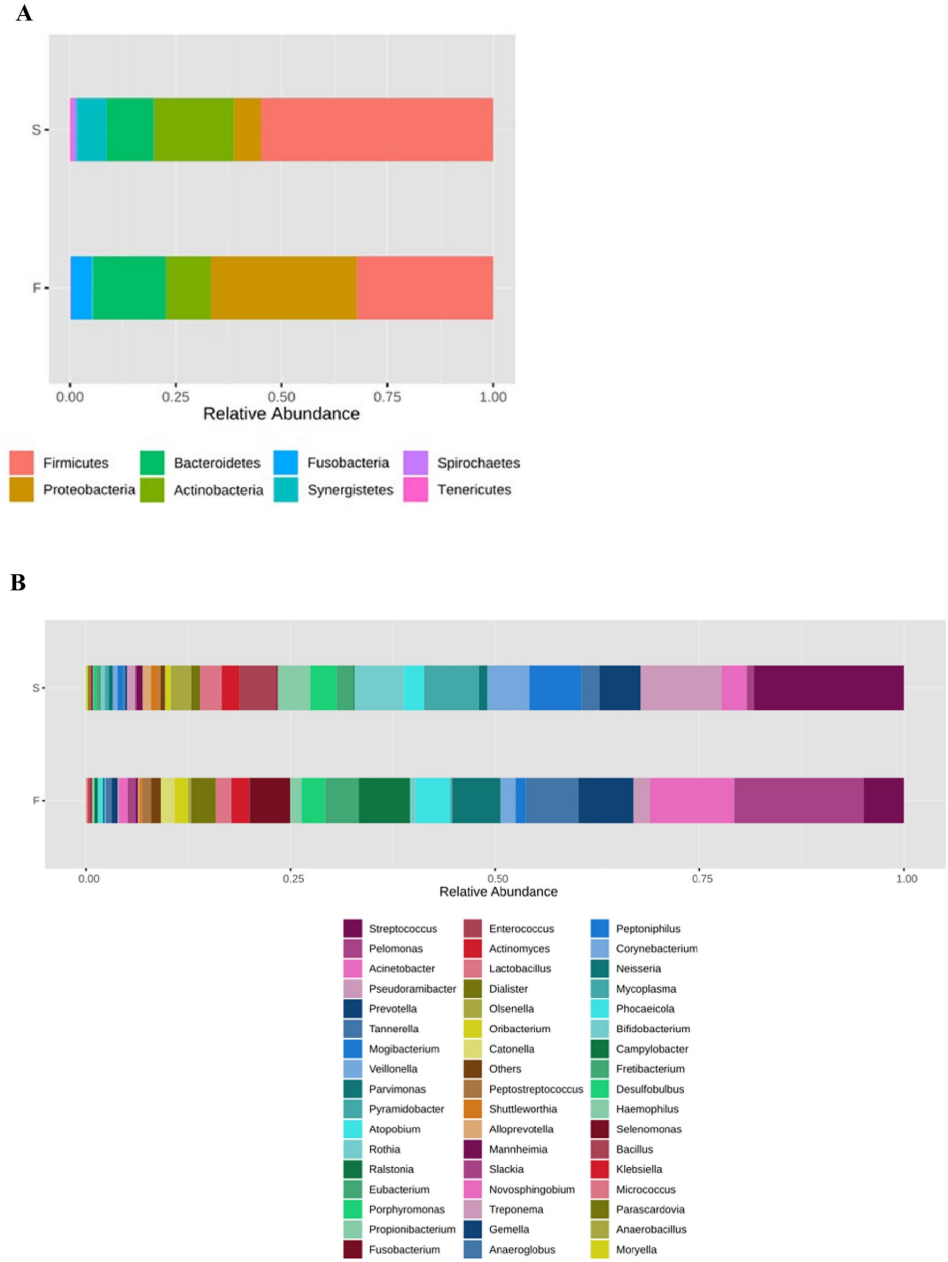
Taxonomic composition at the phylum (A) and genus level (B) using stacked bar chart for primary (F) and post-treatment (S) groups.

The mean and standard deviation (SD) of species-level phylotypes per canal were almost identical; 46.9 ±21.93 for the primary infection group, and 46.8± 16.75 in post-treatment infection samples. In total, eight phyla, 113 genera and 260 species were detected. Fig 1 shows the taxonomic composition at phylum (A) and genus level (B) using stacked bar chart for the two groups. On comparing the taxon compositions of the microbiota in primary and post-treatment infection groups, the most abundant phylum in both groups was *Firmicutes* which was significantly more in the post-treatment (54.4%) than in the primary (32.2%) infections (p>0.05). The other phyla were not significantly different, despite the clear variations. Higher abundance in the post-treatment group was noted for some phyla namely *Actinobacteria* (10.6% and 18.9%), *Bacteroidetes* (11.4% and 17.2%), *Tenericutes* (0.04% and 0.5%) and *Synergistetes* (0.3% and 7.1%), while other phyla were more abundant in the primary infection group including *Proteobacteria* (34.3% and 6.5%), *Fusobacteria* (5.1% and 0.2%), and *Spirochaetes* (0.14% and 0.96%) respectively as shown in Fig 1A.

**Fig 2:**
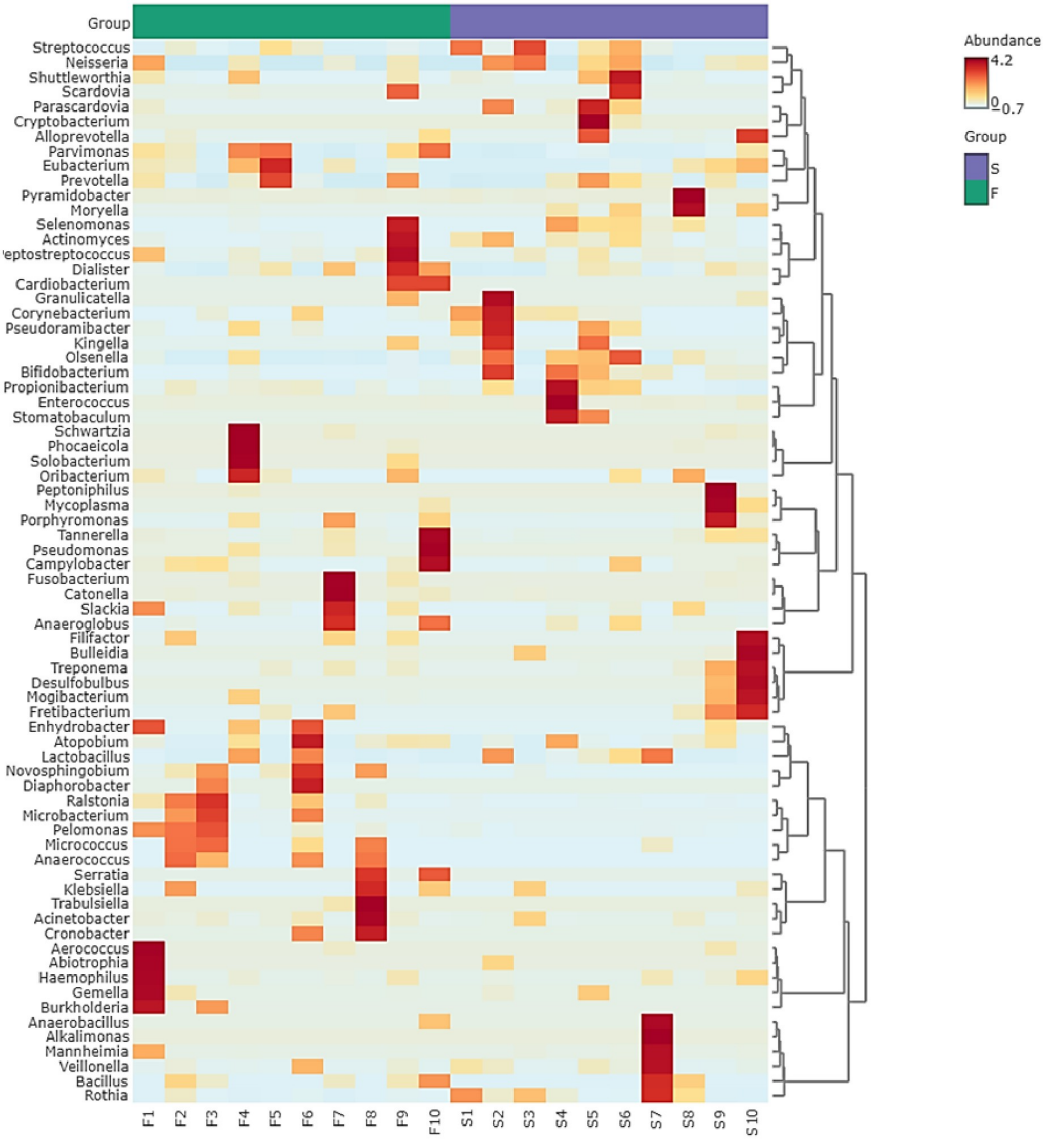
Hierarchical clustering with heatmap and dendrogram (distance measure using Euclidean distance measure and ward clustering algorithm) for the genera detected in each sample.

**Fig 3:**
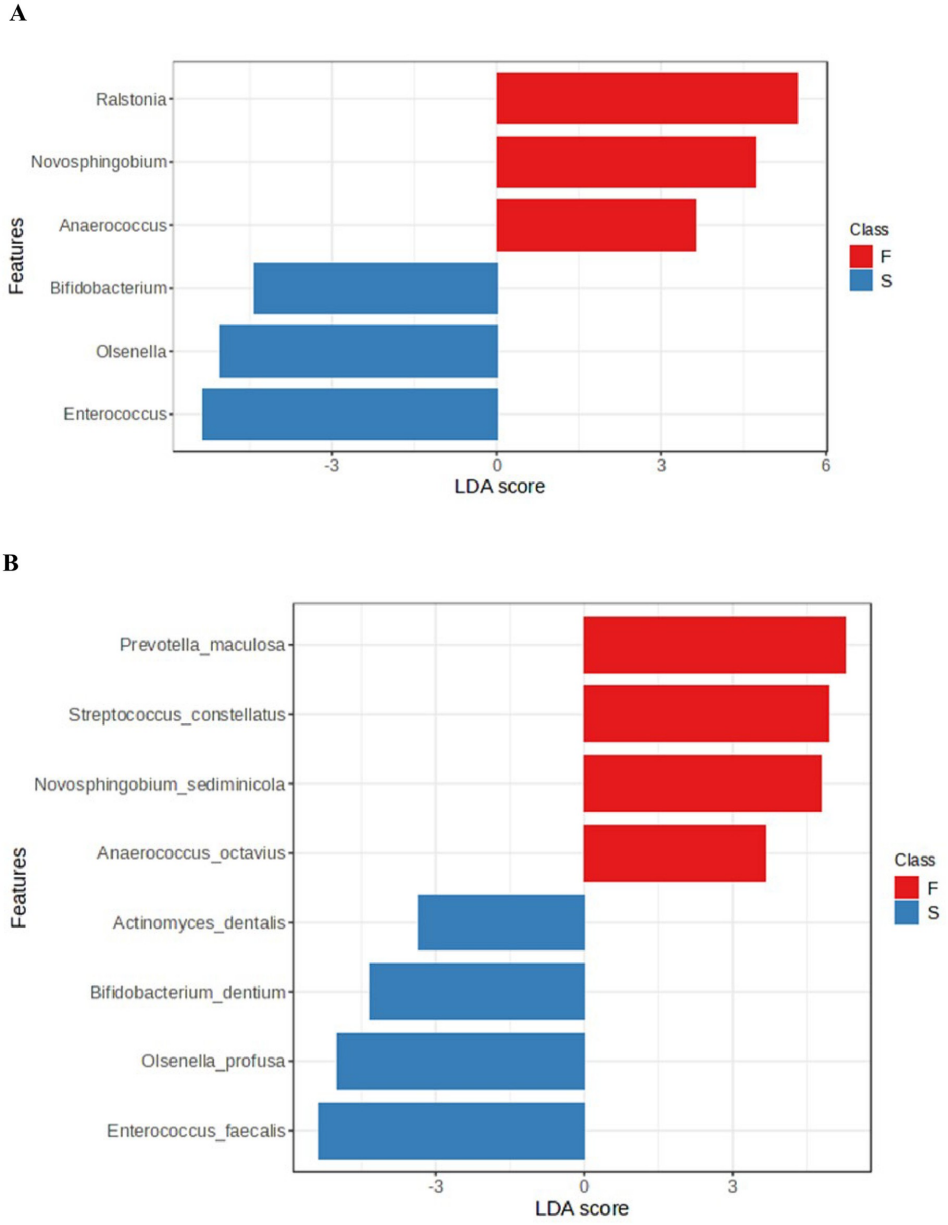
Significant variations in genera (A) and OTUs (B) detected in primary (F) and post-treatment (S) groups identified by linear discriminant analysis (LDA) effect size (LEfSe).

There were disparities in the abundance of genera between the two groups (Figs 1B and 2). Thus *Enterrococcus*, *Bifidobacterium*, and *Olsenella* were significantly more abundant in the post-treatment group, while *Ralstonia*, *Novosphingobium*, and *Anaerococcus*, were significantly more abundant in the primary infection group. LDA analysis, which was used to examine the differential abundance between the two groups, confirmed these significant variations (Fig 3A). Significant differences in the microbiota were also noted at species level (Fig 3B).

*Enterrococcus faecalis* of phylum *Firmicutes* was significantly more abundant in post-treatment (7 out of 10 samples) compared with primary infections (2 out of 10 samples). *Bifidobacterium dentium*, *Olsenella profuse* and *Actinomyces dentalis* were also present in significantly larger quantities in post-treatment infections than the primary infections, while the converse was noted for *Prevotella maculosa*, *Streptococcus constellatus*, *Novosphingobium sediminicola* and *Anaerococcus octavius*. This was also evident by LDA analysis which revealed eight species with significant variations in abundance (Fig 3B).

**Fig 4:**
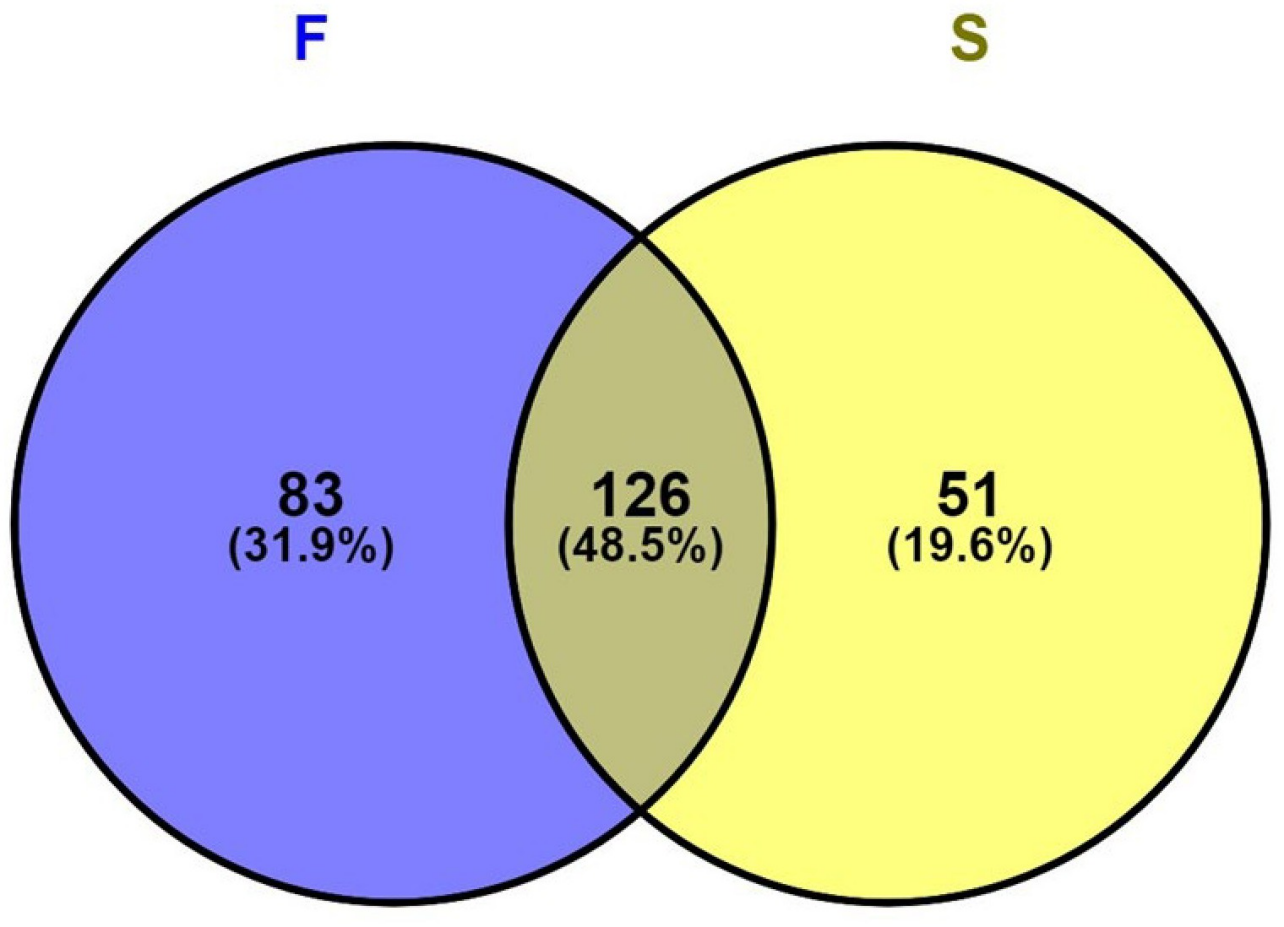
Venn diagram representing the OTUs unique to each type of infection; primary (F) and post-treatment (S), and those shared by both types of infection represented in the overlapping area.

Finally, the OTUs detected in the samples were compared regardless of their relative abundance using a Venn diagram to identify shared and unique species in the two groups (Fig 4). This revealed 83 (31.9%) OTUs unique to primary infection and 51 (19.6%) OTUs only in post-treatment infections. On the other hand, almost half (48.5%, n = 126) of the OTUs were shared between the two groups (Fig 4).

### Diversity analysis between and within groups

**Fig 5:**
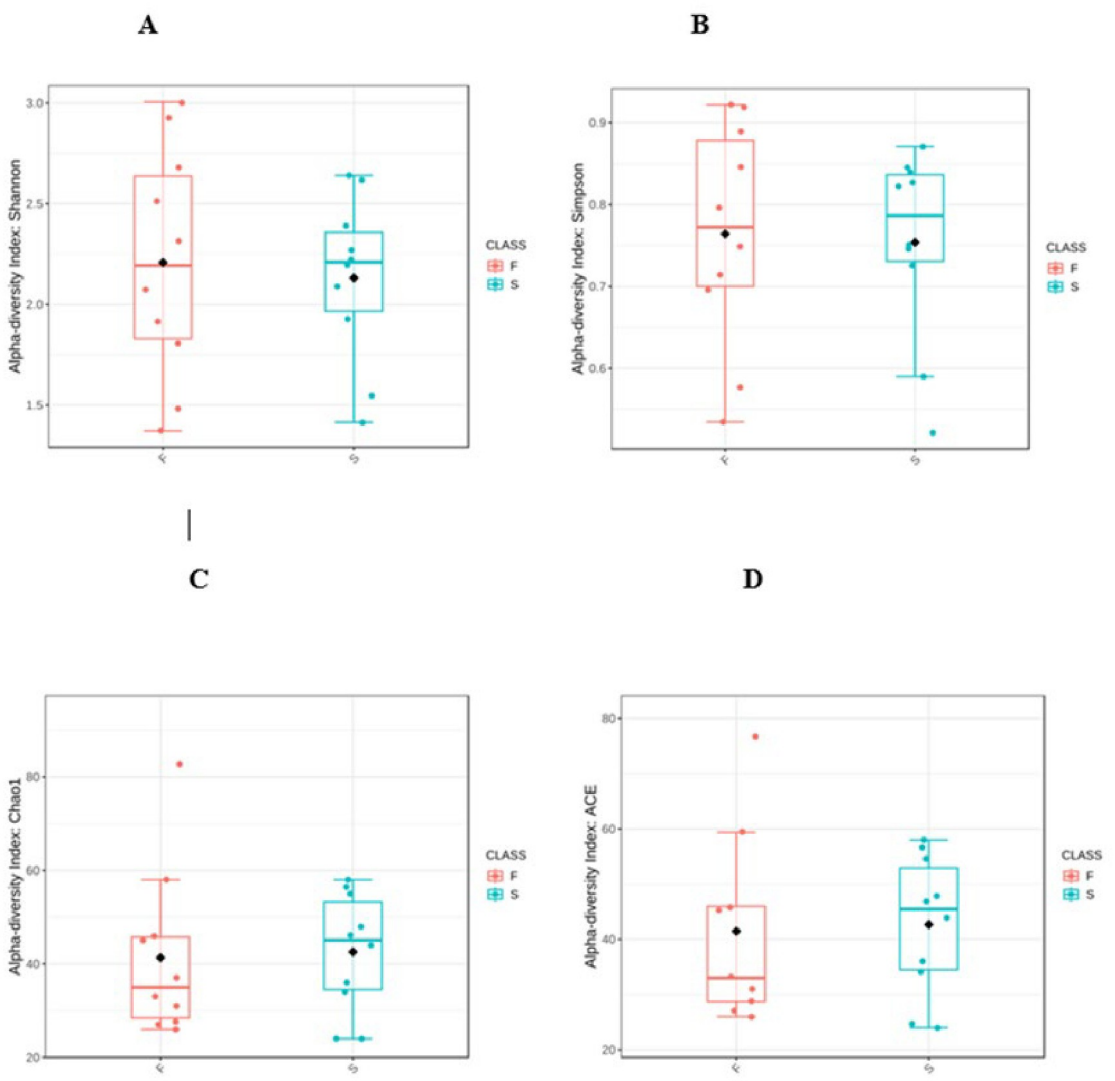
Alpha diversity using different indices in F (primary infection), and S (post-treatment infection) groups represented as boxplots.

The diversity of a sample can be characterized via the total number of species (richness), the abundances of the species (evenness) or measures that consider both the richness and evenness. The Chao1 and ACE measures estimate the richness by inferring the number of rare organisms that may have been lost due to under sampling. Diversity indices such as Shannon and Simpson analyze both the number (richness) and the abundance of organisms (evenness) to describe the actual diversity of a community. Alpha-diversity of our samples were not statistically significant (p>0.05) (Fig 5).

**Fig 6:**
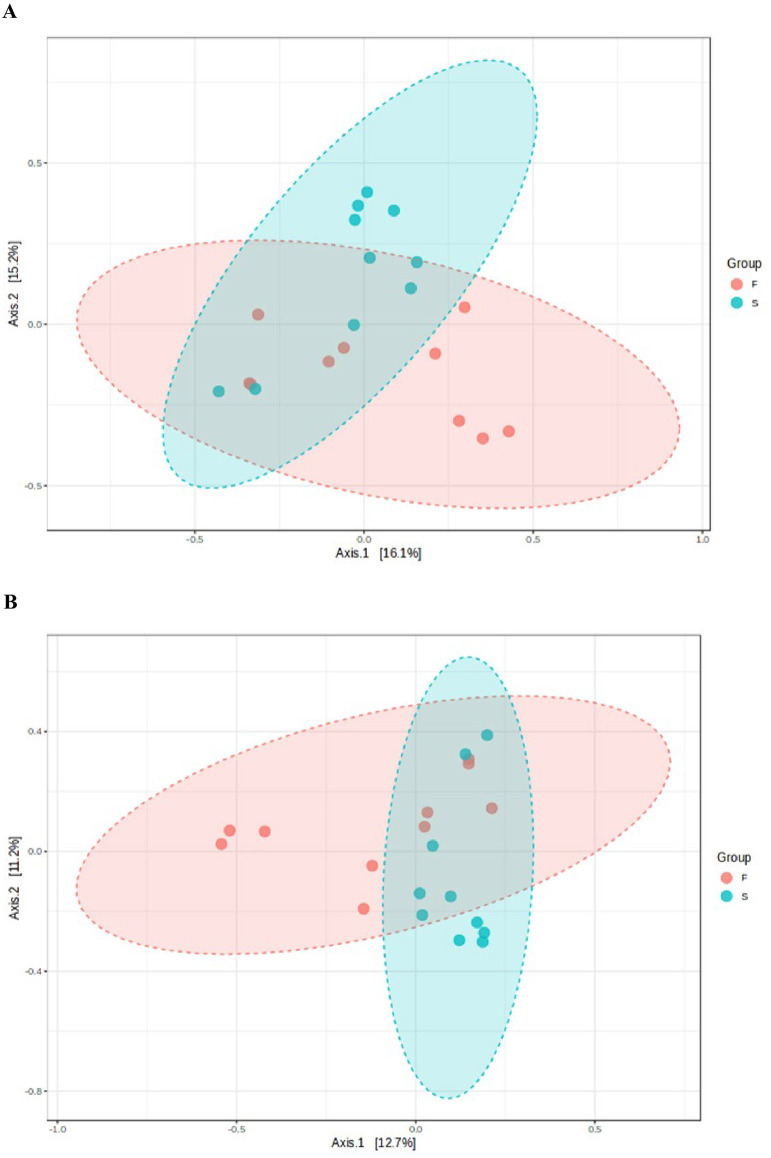
The ordination plot of primary (F) and post-treatment (S) infected samples based on Bray-Curtis distance (A) and Jaccard index (B).

Beta diversity analysis provides a way to compare the diversity or composition between two groups or microbial communities. Bray-Curtis distance and Jaccard index were used as shown in the Principal Coordinate Analysis (PCoA) plots (Fig 6). There was statistically significant difference between the groups based on PERMANOVA (Bray-Curtis distance: F-value: 2.0573; R-squared: 0.10257; p-value: 0.005, and Jaccard index: F-value: 1.6765; R-squared: 0.085203; p-value: 0.003).

**Fig 7:**
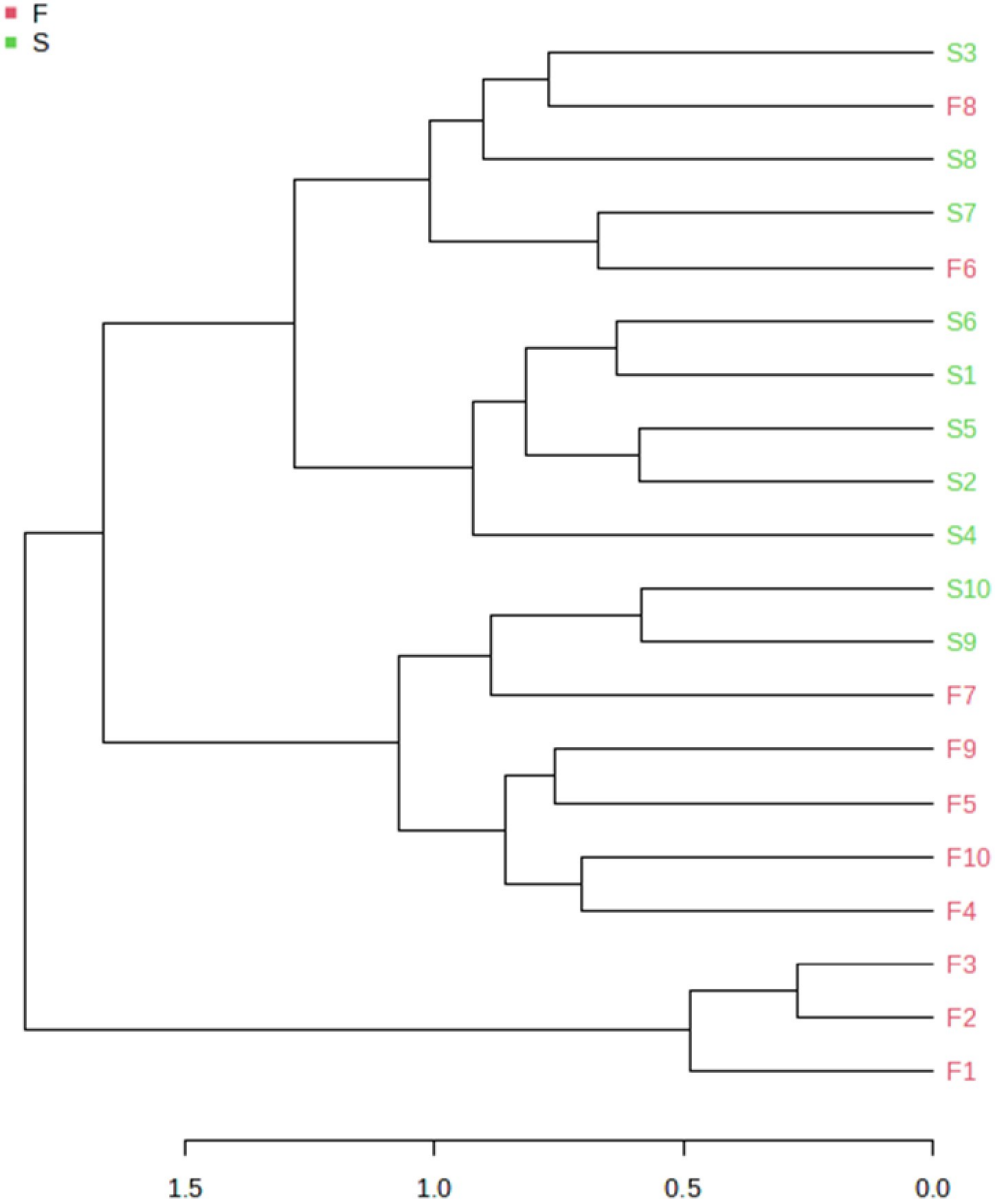
Hierarchical clustering of primary (F) and post-treatment (S) infection samples in the form of dendrogram based on beta diversity.

Based on the beta diversity, hierarchical clustering in the form of a dendrogram is shown in Fig 7. Although some of the samples in both groups showed close clustering indicating similar composition of the microbiota, there were several samples that had distant clusters implying disparate nature of the microbiota between the two groups.

## Discussion

The rationale of the present study was to evaluate the differences, if any, of the microbiota of primary and post-treatment root canal infections in the context of endodontic treatment. Several studies have used NGS to decipher the bacteriome of the infected root canals and these were used as reference points for the current investigations [7, 21].

With regards to the number of species detected per root canal, the mean number of species-level taxa in each canal was almost identical with 46.9 ±21.93 and 46.8± 16.75 in primary and post-treatment infections, respectively. Siqueira et al., (2011) have reported similar findings with a mean number of 37 species level taxa (range 13–80) in the apical section of the root canal from primary infected extracted teeth [22]. On the contrary Ozok et al. (2012) reported a much higher mean of 125 taxa per canal in primary infected extracted canals [23] while others have described much lower, 10–20 species [24, 25]. Nonetheless, the sampling and analytical techniques in the latter studies are unclear and inconclusive. Clearly, therefore, sampling quality is a major factor impacting the outcome of such studies.

In the current study, eight phyla, 113 genera and 260 species were identified using NGS technology. Firmicutes was the most abundant phylum identified in both the primary and post treatment infections, comprising more than one third of the constituents (37.6%), followed by *Proteobacteria* (26%), *Actinobacteria* (15.7%), *Bacteroidetes* (11.9%), *Fusobacteria* (3%), *Spirochaetes* (3%), *Tennericutes* (0.76%) and *Synergistetes* (0.76%). In agreement with our study, Bouillaguet et al., (2018) and Qian et al. (2019) have previously reported *Firmicutes* as the most abundant phylum in similar lesions [13, 26]. In contrast, others have variously reported *Bacteroidetes*, and *Proteobacteri*a as the most abundant phyla [8, 27–30], again indicating that analytical techniques may account for such differences.

The predominant genera identified in primary infections in the present study were *Prevotella*, *Fusobacterium*, *Lactobacillus*, *Acinetobacter*, *Burkholderia*, *Campylobacter*, *Pseudomonas*, *Corynebacterium*, *Veillonella*, *Mogibacterium* and *Treponema*. Some, but not all, of these genera have been previously identified through NGS analysis in primary infections by a number of workers [22, 23, 31].

The dominant genera identified in the present study in post-treatment, as opposed to primary infections were *Prevotella*, *Treponema*, *Lactobacillus*, *Streptococcus*, *Leptotrichia*, *Aggregatibacter*, *Bifidobacterium*, *Proprionibacterium* and *Mycoplasma*. Additionally, there were also significant variations in three genera namely *Enterococcus*, *Bifidobacterium* and *Olsenella* in post-treatment infections when compared to primary infections. Yet again previous reporters have recorded most of these genera in various proportions in post-treatment infections [32–34].

The genus *Enterococcus* frequently detected in root filled teeth with infection is classically regarded as a major endodontic pathogen, even prior to the NGS era [32, 35, 36]. Indeed, we noted that *E*. *faecalis*, was more prevalent in post-treatment cases than in primary infections confirming the notion that it is a secondary opportunistic colonizer in treated root canals rather than a persister from unsuccessfully treated primary infections [2, 37, 38].

Other major genera detected in the current study includes *Bifidobacterium*, a major organism in our study, which is a significant genus in primary infections and have shown an intimate association with the carious process [39] as well as de novo [40] and refractory apical periodontitis [41]. It is therefore probable that they play an important role in extension of the carious process into an endodontic infection. *Olsenella* species are obligatory anaerobes [42]. Previous studies have reported them as key pathogens in endodontic infections in association with *O*. *uli* [43, 44]. Interestingly, the latter was not one of the most abundant species we noted.

Intriguingly, we identified *Ralstonia* genus, an aerobic, non-fermentative, Gram-negative bacillus commonly found in water and soil in our studies together with *Novosphingobium* which are metabolically versatile, as reported by many in previous studies [21, 29, 45]. The reason for their presence in endodontic lesions is yet to be determined.

As regards the diversity and richness of the samples, although the alpha diversity values which measure the richness and evenness of the OTUs in the primary and post-treatment infections were not significantly different, we noted significant difference in beta diversity in these infections (p<0.05). This is not surprising as the eco-biome of the root canal is vastly different in primary infections relative to post-treatment infections after radical mechanical and chemical interventional procedures. Such bacterial diversity in primary and post-treatment infections have been previously noted by Buonavoglia et al., [46] and Ordinola-Zapata R. et al., [47].

As seen above, the diversity in the microbial composition of infected root canals could be due to true geographical reasons. Indeed, de Oliveira et al., (2007) and Zehnder & Guggenheim, (2009) have shown that the indigenous water quality or ecologic factors profoundly impact the quality of the oral microbiota [38, 48]. All of the above-mentioned studies apart from this study were not from the Middle Eastern/Gulf region and were from an assortment of geographical locales. Hence the present study is a first in this context from UAE though the data needs to be confirmed by further investigations.

In addition, specific intrinsic factors appertaining to the ecosystem of the root canal environment which is constantly changing during the progression of an infection may account for the variations in the microbiota. These include the intracanal pH or the redox potential that is constantly changing, and the concomitant tissues necrosis as the lesion progresses [49]. Therefore, sampling root canals at different stages of infection may result in significant variations in both the qualitative as well as the quantitative yield of the microbiota.

Other major sources of variation in results between studies could be the different irrigates and disinfectants used in intracanal disinfection, and the sampling technique employed by various workers in harvesting the microbiota. Additionally, discrepant results between studies could also be due to complex NGS workflows in which potential biases may occur during DNA extraction, PCR amplification, sequencing, or bioinformatic analysis pipelines [50, 51]. Finally, as DNA based studies cannot differentiate dead from live organisms, there is a possibility of exaggerated bacterial load being reported [40]. However, some have argued that all bacteria present in the endodontic space could have played a role in the different phases of the disease at one time or another [52].

For all these reasons, it appears that until the advent of further refinements in sampling and analytical technology of endodontic microbiome, it is useful to integrate data derived from both the traditional, cultural methods as well as the novel NGS technology, to yield a more realistic profile of the diversity of the endodontic flora.

While acknowledging the constraints posed by the limited sample size, our findings offer valuable insights into the microbiome composition of endodontic infections. We elucidated notable distinctions between primary and post-treatment cases, underscoring the need for additional investigation in this realm. The current findings testify to the complex, polymicrobial etiology of endodontic infections and the need for further large-scale clinical studies with validated standardized protocols for improved understanding of the pathogenesis and management of endodontic infections.

## Conclusion

Analysis of 16S rRNA gene sequences for infected root canals revealed the microbiota in primary and post-treatment infections. There were significant differences in microbiota composition and diversity between the groups, however, the majority of the taxa detected were in low abundance. Although the bacterial communities were more diverse in primary infections, it was not statistically significant when analyzing the alpha diversity, but there was a statistically significant difference in beta diversity when comparing the primary and post- treatment infections groups inferring that there was inter-individual variability. The use of NGS technology can aid in the identification of key microbiota implicated in the pathogenesis of endodontic infections and can guide the management of these infections. Notably, a dearth of knowledge exists regarding the characterization of endodontic microbiota within the United Arab Emirates (UAE) and Gulf populations [53], underscoring the novelty and significance of our study. These findings not only enhance our understanding of local microbial profiles but also pave the way for future research endeavors aimed at filling this critical gap in our knowledge.

## Supporting information

S1 Data (XLS)
